# Flexible Multiplane Structured Illumination Microscope with a Four-Camera Detector

**DOI:** 10.3390/photonics9070501

**Published:** 2022-07-20

**Authors:** Karl A. Johnson, Daniel Noble, Rosa Machado, Tristan C. Paul, Guy M. Hagen

**Affiliations:** UCCS BioFrontiers Center, University of Colorado Colorado Springs, 1420 Austin Bluffs Parkway, Colorado Springs, CO 80918, USA

**Keywords:** structured illumination, fluorescence, brain, multicamera

## Abstract

Fluorescence microscopy provides an unparalleled tool for imaging biological samples. However, producing high-quality volumetric images quickly and without excessive complexity remains a challenge. Here, we demonstrate a four-camera structured illumination microscope (SIM) capable of simultaneously imaging multiple focal planes, allowing for the capture of 3D fluorescent images without any axial movement of the sample. This setup allows for the acquisition of many different 3D imaging modes, including 3D time lapses, high-axial-resolution 3D images, and large 3D mosaics. We imaged mitochondrial motions in live cells, neuronal structure in *Drosophila* larvae, and imaged up to 130 μm deep into mouse brain tissue. After SIM processing, the resolution measured using one of the four cameras improved from 357 nm to 253 nm when using a 30×/1.05 NA objective.

## 1. Introduction

Structured illumination microscopy (SIM) is a technique in fluorescence microscopy in which sets of images are acquired with shifting illumination patterns; subsequent image processing of these image sets can yield results with optical sectioning, resolution beyond the diffraction limit (super-resolution), or both [1–6]. Since its emergence over two decades ago [7], SIM has matured significantly as an imaging technique, with multiple proposed methods for generation of the SIM pattern [4–17], as well as for processing of the image data [3,6,18–22]. SIM has higher resolution and better optical sectioning abilities than confocal microscopy or spinning disc confocal microscopy [6,12], while also potentially being less expensive to implement. Compared to other super-resolution techniques, the speed, high signal-to-noise (SNR) ratio, and low excitation light intensities characteristic to SIM make it ideal for the imaging of live samples in 3D.

Producing a 3D image with fluorescence microscopy requires intensity information throughout the sample to later be localized to a specific location in 3D space. With SIM, this is traditionally accomplished by acquiring a series of optically sectioned images of the sample while translating the sample axially through the focal plane of the microscope (a *Z*-stack). This method yields discrete *XY* slices of the sample, which can then be combined into a 3D image. Although this sequential approach remains a staple of fluorescence microscopy, the substantial light exposure to the sample, long acquisition times, and possible agitation of the sample incurred by this method are drawbacks.

As such, several other methods have been developed as an alternative to this traditional approach. Prominent among these is multifocal plane microscopy, in which multiple focus-shifted image planes are imaged simultaneously, removing the need for sample movement. This can be achieved using a variety of techniques; in recent years, multiple studies have demonstrated several methods to image multiple image planes side by side onto a single detector. Two of the approaches to achieve this involve the use of a multifocal grating [23–27] or a variable-path-length image-splitting prism [28,29]. The specialized optical elements central to these approaches are difficult to design and can restrict the versatility of the resulting setups. Since both approaches have a defocus distance defined by the physical features of the optics being used, the distance between imaging planes in the sample cannot be easily adjusted. Both of these optical systems are designed to be used with a particular microscope objective, which cannot be changed without redesigning the multifocal optics. Such limitations limit the usefulness of these previously reported systems.

Recently, Xiao et al. proposed a reconfigurable multiplane microscope which uses an array of beam-splitters to image multiple focal planes on a single detector [30]. Unlike the aforementioned methods, this approach is compatible with a variety of microscope objectives. Nevertheless, this approach requires the construction of a ‘z-splitter’ assembly, and the separation between image planes produced by this optical system cannot be modified without changing critical dimensions of the ‘z-splitter’.

Multicamera approaches have also previously been explored in fluorescence microscopy as a method for multifocal plane microscopy. These methods are particularly attractive due to their optical simplicity and versatility. In 2004, Prabhat et al. proposed an optical system in which two cameras are placed at different focal positions relative to the microscope’s tube lens, resulting in each camera capturing an image at a different focal plane in the sample [31]. Others have since expanded upon this idea by using this multidetector method for 3D localization microscopy [32] or by expanding the number of detectors to three or four [32,33].

Unfortunately, the viability of multicamera imaging is limited by the high cost of scientific cameras. Recently, complementary metal–oxide–semiconductor (CMOS) sensor technology has improved significantly in terms of both performance and price, yielding a variety of affordable cameras with sufficient sensitivity and noise performance for fluorescence microscopy. In 2018, Babcock demonstrated a 3D localization microscopy system using four machine vision-grade CMOS cameras [32]. The cameras could detect the extremely dim signals necessary for localization microscopy (10–1000 photons/pixel) while costing an order of magnitude less than typical scientific CMOS (sCMOS) cameras.

The usability of multicamera imaging for SIM is complicated by the adverse effects of placing detection planes at focal positions defocused from the illumination plane of a 2D SIM pattern. For one, the contrast of high-spatial-frequency illumination patterns deteriorates rapidly as the defocus of the detection planes increases. Increased defocus distances can also introduce undesired artefacts such as varying magnification (if the system is not telecentric) and defocus distances which depend on the refractive index. In this study, we characterize some of the adverse effects of focal plane multiplexing in a 2D SIM system and provide methods to overcome these issues.

We demonstrate four different imaging modes achievable using a four-camera detection system in conjunction with SIM methods. Switching between modes can be performed without physical manipulation of the optical setup. This allows for versatility in imaging, as different types of samples are best imaged by different modes. As live cells constantly move, they are best imaged by taking a sequence of 3D images over time, yielding a 3D movie of the cell. For static samples with a significant axial extent, a *Z*-stack of 3D images can be acquired, producing a high-axial-resolution 3D image but with a fourfold reduction in sample movements. Larger samples can be imaged by taking a mosaic of 3D images, resulting in images with a large field of view (FOV) and useful axial information. We also performed multicolor imaging with the same setup. Image processing for all these imaging modes can be performed using our open-source software for SIM (SIMToolbox) [34] along with standard tools in MATLAB (The MathWorks, Natick, MA, USA) and ImageJ [35].

## 2. Materials and Methods

### 2.1. Microscope Setup and Data Acquisition

This project uses a home-built SIM setup based on the same design described in our previous publications [12,18,36,37]. The current SIM system is based on an IX83 microscope (Olympus, Tokyo, Japan) with the four-camera setup serving as the detector. The data in this study were collected using UPLSAPO 30×/1.05 NA silicone oil immersion and UPLSAPO 60×/1.35 NA oil immersion objectives (Olympus, Tokyo, Japan), although our setup is compatible with any objective meant to be used with the IX83 microscope. Sample movement was controlled with an ASI motorized *XY*, piezo *Z* stage (Applied Scientific Instrumentation, Eugene, OR, USA). We used a quadruple-band fluorescence filter set (part 89000, Chroma, Bellows Falls, VT, USA). To synchronize the four cameras with SIM illumination and stage movement, we used Andor IQ software (Belfast, UK).

Our SIM system uses a ferroelectric liquid crystal on silicon (LCOS) microdisplay (type SXGA-3DM, Fourth Dimension Displays, Dalgety Bay, Fife, UK). The LCOS microdisplay has been utilized before in SIM and related methods in fluorescence microscopy [2,12,18,34,38–41], and it allows 2D patterns of illumination to be projected onto the sample that can be reconfigured by changing the image displayed on the device. The light source (Lumencor Spectra-X, Beaverton, OR, USA) is toggled off between SIM patterns and during camera readout to reduce unnecessary light exposure to the sample. Since this system uses a microdisplay to generate the SIM patterns, the spatial frequency, angle, and number of phases comprising the SIM pattern can be reconfigured at any time. This flexibility allows for optimization of the imaging conditions for each experiment, responsive to the properties of the sample and the defocus between detection planes. We used a PM100D optical power meter with S12C detector (Thorlabs, Newton, NJ, USA) to measure the excitation power at the back aperture of the objective. We measured less than 100 μW of optical power in all cases, with the exact measurements being given in Supplementary Table S1.

For detection, our setup uses four Blackfly-S USB 3.0 machine vision cameras. (model: BFS-U3-31S4M, FLIR, Arlington, VA, USA). The manufacturer’s specifications for some of the relevant parameters of this camera are shown in Table 1. These cameras have acceptable parameters for fluorescence microscopy such as 62% quantum yield and low noise, but at a far lower price point than the sCMOS cameras typically used. The signal-to-noise ratio of these cameras was compared to an Andor Zyla 4.2+ sCMOS camera. The results are shown in the Supplementary Materials. As expected, the machine vision cameras had poorer performance compared to the Andor sCMOS camera; however, the performance of these cameras was adequate for the purposes of this study. The machine vision cameras have a smaller pixel size, which allows for Nyquist sampling to be achieved at lower magnifications than when using the Zyla. We note that CMOS sensors are being developed rapidly and that many other camera models are currently available. Other machine vision cameras may have better specifications at higher prices.

**Figure 1.**
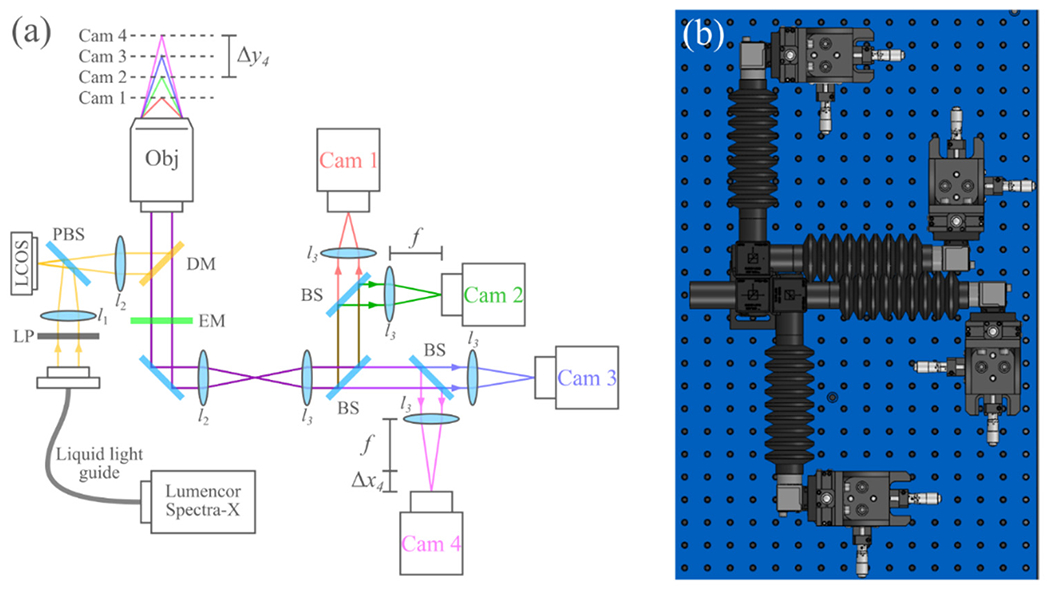
(**a**) Overview of the optical system. Δx_4_ and Δy_4_ indicate the sensor defocus and sample defocus of camera 4, respectively. LP, linear polarizer; LCOS, liquid crystal on silicon microdisplay; PBS, polarizing beam-splitter; DM, dichroic mirror; EM, emission filter; BS, 50–50 beam-splitter; l_1_, 50 mm FL; l_2_ 180 mm FL Olympus tube lens (part SW-TLU); l_3_, 175 mm FL. (**b**) To-scale model of the four-camera detection system.

In principle, the SIM and detection system in this study can acquire raw SIM frames at a rate up to the maximum frame rate of the cameras. Since four cameras are capturing such images simultaneously, this could allow for ~220 Hz acquisition of raw SIM frames. In practice, the 100 ms+ exposure times needed for the samples in SIM limit the system to a raw frame rate of ~40 Hz and a reconstructed frame rate of ~3–7 Hz (depending on the number of phases in the illumination pattern). The four cameras were mounted on *XYZ* translation stages (MT3, Thorlabs, Newton, NJ, USA) to enable precise positioning of each camera. SpinView software (FLIR, Wilsonville, OR, USA) was used to acquire the images. Figure 1 shows a diagram and 3D model of the optical system.

In order to image multiple distinct planes in the sample, each camera was placed at a slightly different distance from each camera’s respective relay lens (lenses l_3_ in Figure 1). Importantly, the light paths of all four cameras are based on a 4*f* design. This design ensures near telecentricity on all four cameras, and as such, defocusing the detectors results in nearly no change in magnification. To position the cameras appropriately, we first aligned all cameras at the focal length of each camera’s respective l_3_. Then, each camera was displaced using its translation stage to achieve a particular defocus. The relationship between this image sensor defocus Δ*x_n_* and the defocus of the sensor conjugate (focal plane) in the sample Δ*y_n_* for each camera *n* can be described by

(1) $$\text{Δ}y_{n}=\frac{\eta_{sample}}{M^{2}}\text{Δ}x_{n},$$

where *M* is the magnification of the optical system, and η_*sample*_ is the refractive index of the sample. This approach is valid for real (noncomplex) refractive indices. In our setup, we positioned each camera such that the focal planes were evenly spaced by a slice spacing Δ*s*. Additionally, to reduce aberrations caused by excessive defocus distances from the focal plane of the SIM pattern, cameras were defocused both forward and backward, with camera 2 kept stationary. This yields the following formulae for the translation stage displacements of each camera, given the desired slice spacing Δ*s*:

(2) $$\text{Δ}x_{1}=\frac{-M^{2}}{{\text{η}}_{sample}}\text{Δ}s.$$

(3) $$\text{Δ}x_{2}=0.$$

(4) $$\text{Δ}x_{3}=\frac{M^{2}}{{\text{η}}_{sample}}\text{Δ}s.$$

(5) $$\text{Δ}x_{4}=\frac{2M^{2}}{{\text{η}}_{sample}}\text{Δ}s.$$

To verify the relation in Equation (1), 100 nm fluorescent beads (F8800, ThermoFisher Scientific, Waltham, MA, USA) dried on a coverslip and mounted with Prolong Glass (ThermoFisher Scientific, Waltham, MA, USA η = 1.518) were imaged using SIM illumination with all four cameras and a 30×/1.05 NA silicone oil immersion objective. To acquire volumetric data of the bead images on each camera, the *Z* stage was scanned over 10 μm in 100 nm increments. Due to the refractive index mismatch between the sample (η = 1.518) and the silicone microscope oil (η = 1.406), the actual spacing between *z*-planes in the final image must be adjusted using a correction factor [42].


(6) $$\sqrt{\frac{{\text{η}}_{2}^{2}-{\text{NA}}^{2}}{{\text{η}}_{1}^{2}-{\text{NA}}^{2}}}.$$

**Figure 2.**
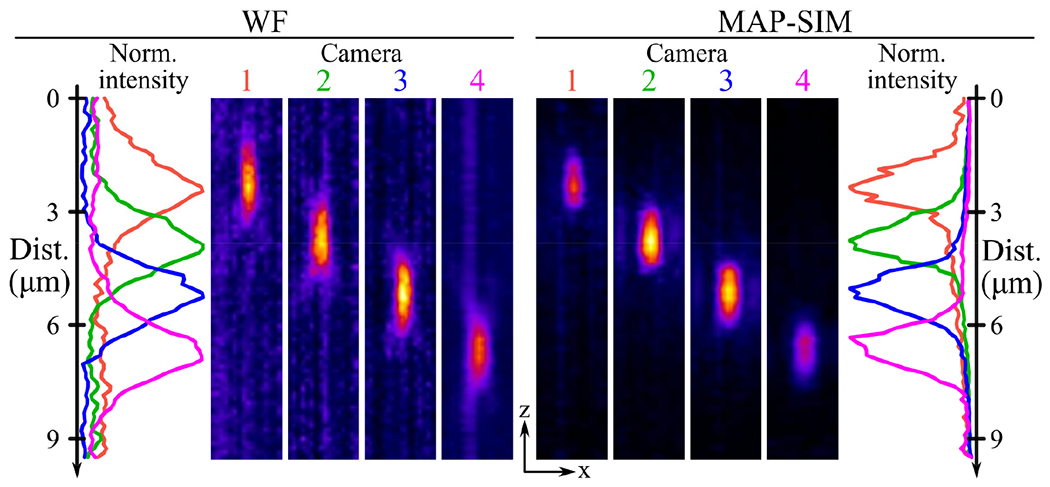
The 100 nm fluorescent nanobead imaged using SIM with defocused detectors. The left side of the figure shows the axial intensity profile and orthogonal projections of the widefield reconstruction. The right side of the figure shows the same for the MAPSIM reconstruction of the data. In both reconstructions, the displacement between the image of the nanobead aligns very well with the predicted focal plane displacement of 1.41 μm. Additionally, the improved axial resolution of the MAPSIM reconstruction is visible in the intensity profile plots.

In this scenario, applying this correction factor yields an actual slice spacing of 117 nm. With this correction, the orthogonal projections of the resulting data, shown in Figure 2, indicate that the bead’s images are displaced axially by an amount close (<5% error) to that predicted by Equation (1). Here, each camera was displaced in 0.9 mm increments, and the measured magnification of the system was 30.86×. Using these values with Equation (1) yields a predicted slice increment of 1.43 μm in the sample. This aligns very well with the average slice spacing measured from the data in Figure 2 (1.4 μm) and fairly well with the individual displacements between focal planes in the same data (1.42, 1.36, and 1.46 μm). Additionally, the measured axial resolution of the MAPSIM reconstruction in these data (1.24 μm full width at half maximum (FWHM)) was ~25% better than that of the widefield reconstruction (1.66 μm).

### 2.2. Cell Lines and Reagents

Hep-G2 cells were maintained at 37 °C and 100% humidity in DMEM supplemented with 10% fetal bovine serum, 100 U/mL penicillin, 100 U/mL streptomycin, and l-glutamate (Invitrogen).

### 2.3. Preparation of Samples for Imaging

Hep-G2 cells were grown in coverslip-bottom imaging dishes for 48 h, and then labeled with Mitotracker Red CMXROS (M7512, ThermoFisher) according to the manufacturer’s recommended protocol. Briefly, cells were labeled with 1 mM Mitotracker for 30 min at room temperature, and then washed twice with and imaged in phosphate-buffered saline, pH 7.4.

We also imaged third-instar *Drosophila melanogaster* (ppk-CD4-tdTom) larvae, which express CD4-tdTomato in the sensory neurons. We anesthetized the larvae using isoflurane. The larvae were then placed on a chamber slide with a drop of SlowFade Gold antifade mountant (S36936, ThermoFisher Waltham, MA, USA), and sealed with a #1.5 coverslip.

We imaged a GFP-labeled mouse brain sample acquired from the SunJin Lab Company (Hsinchu City, Taiwan). This sample is a 250 μm thick coronal section which was cleared and mounted by SunJin Lab using their RapiClear 1.52 reagent.

Lastly, we demonstrated multicolor imaging of a slide (FluoCell prepared slide #1, ThermoFisher Scientific, Waltham, MA, USA) in which bovine pulmonary artery endothelial cells (BPAE cells) were labeled with MitoTracker red CMXROS (labels mitochondria) and Alexa Fluor 488 phalloidin (labels actin).

### 2.4. Image Preprocessing

Immediately after acquisition, hot pixels appear throughout most of the raw image data, as the industrial CMOS cameras used in this study are not actively cooled and were operated at room temperature. Since hot pixels can cause undesirable artefacts in our super-resolution SIM reconstruction algorithm, hot-pixel removal is necessary prior to SIM reconstruction. To do this, we collected dark images with each of the cameras using the same exposure settings as in the image data, and then subtracted these dark images from each frame of the raw data. While this simple method is only effective on the hot pixels which appear in both the dark frames and the raw data, these hot pixels make up the vast majority of the total number of hot pixels in the raw data; as such, this method is satisfactory.

### 2.5. SIM Data Processing

SIM reconstructions were performed as previously described using SIMToolbox, an open-source and freely available program that our group developed for processing SIM data [34]. We generated optically sectioned, enhanced-resolution images using an established Bayesian estimation method, maximum a posteriori probability SIM (MAPSIM) [18,36,37]. Widefield (WF) and conventional resolution, optically sectioned (OS-SIM) images were also reconstructed using SIMToolbox.

### 2.6. Image Registration and Assembly/Stitching

After SIM reconstruction, it is necessary to digitally align the images from the four cameras, as it is not practical to perfectly align the images from each camera using the translation stages. First, a single processed frame is selected from each camera to perform image registration. After selecting one of the cameras as a reference, all other images are registered to the reference camera’s image using a gradient descent optimization method. This registration process yields an alignment matrix for each camera which describes the transformation necessary to transform each camera’s image to align with the reference camera’s image. Next, all processed images from the nonreference cameras are transformed using the alignment matrices found during registration, yielding images that are aligned to the reference. This image registration and transformation process was performed using the image processing toolbox for MATLAB.

**Figure 3.**
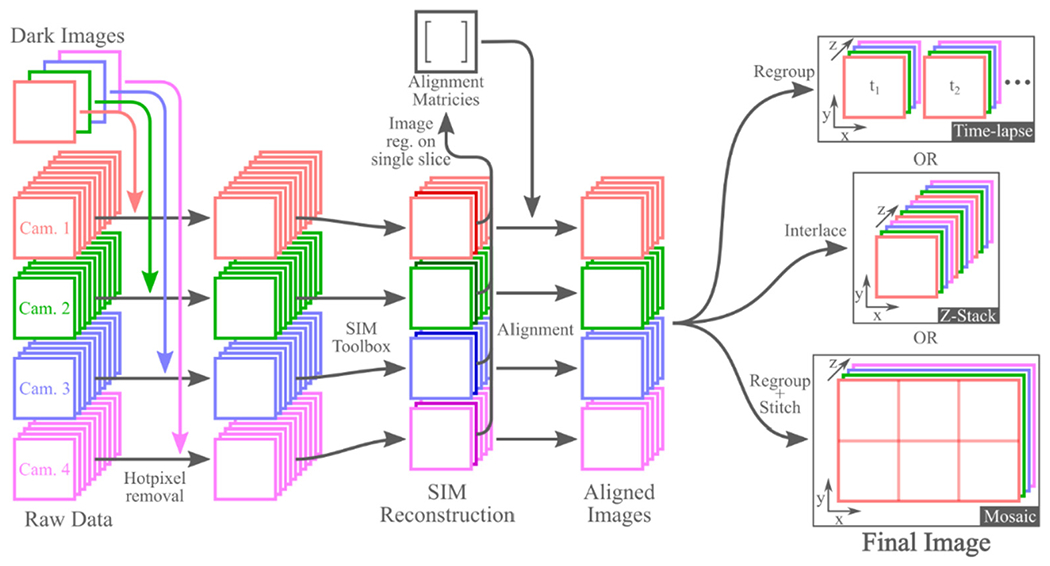
Overview of the standard processing procedure for all three imaging modes. Note that the hot-pixel removal and image registration steps only must be performed once per dataset, and they do not have to be repeated when doing processing for each subsequent SIM reconstruction method. Additionally, while this figure shows the image registration as being performed on a single slice of the reconstructed SIM data, these are not the only images on which registration can be performed. Registration can also be performed on a maximum intensity projection of each camera’s reconstructed data or on images of a calibration slide.

After alignment of the images from each camera, the data are organized into four separate image stacks (one for each camera). The process to reconfigure these data into their final form is dependent on the imaging mode. For 3D time-lapse imaging, the four image stacks can be regrouped into multiple image stacks, one for each time period. For Z-stacks of 3D images, interlacing the four image stacks into a single image stack produces the final 3D image. To generate 3D mosaics, the four camera image stacks must be first regrouped into multiple image stacks, each containing one 3D image tile. Similar to the time-lapse mode, each of these image stacks will contain four slices, one from each camera. These 3D images can then be stitched into the final image. We performed image stitching using Preibisch’s plugin for ImageJ [43]. The entire data processing procedure is summarized in Figure 3.

## 3. Results

**Figure 4.**
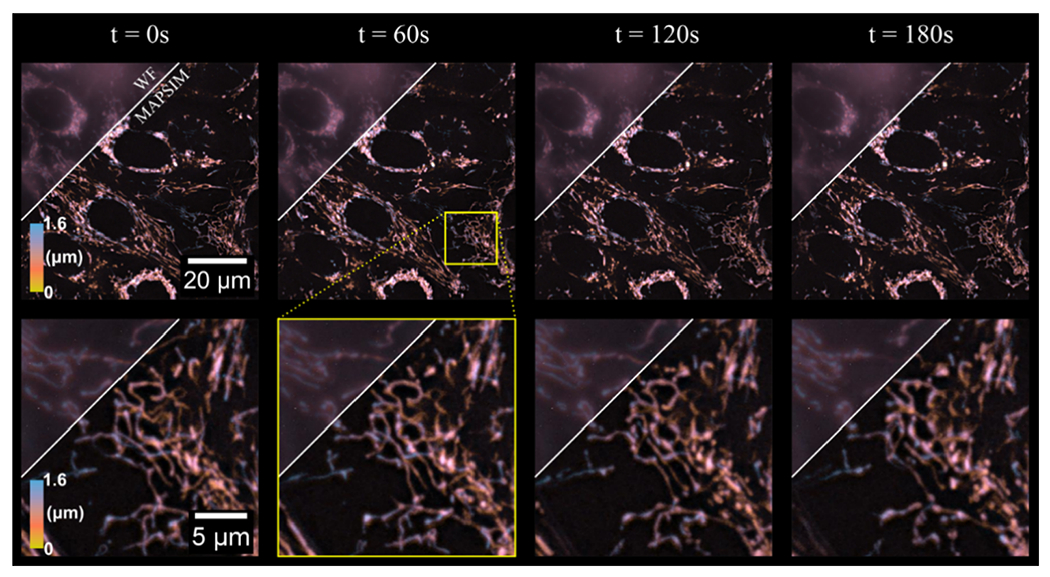
The 3D time lapse of mitochondrial dynamics in HEP-G2 cells. The region above the diagonal line on each frame shows the WF reconstruction of each time frame, with the region below the line showing the MAPSIM reconstruction of the data. Objective: UPLSAPO 60×/1.35 NA oil immersion. Imaged at 37 °C and 5% CO_2_ using type 37 oil (Cargille).

**Figure 5.**
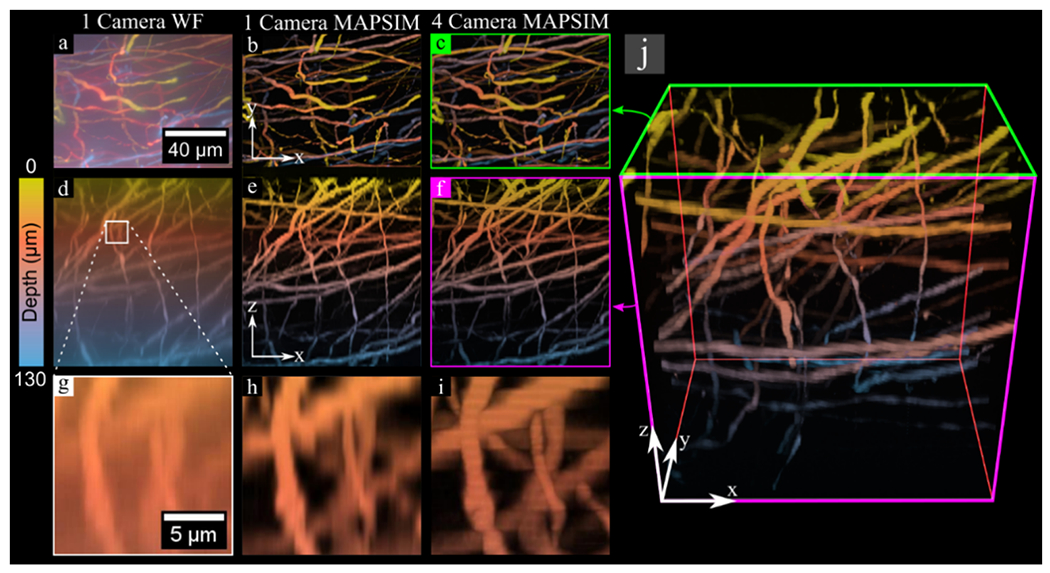
The 3D image of neurons in a GFP-labeled mouse brain, acquired using the *Z*-stack imaging mode. All data were color-coded according to the scale bar at the left of the figure. (**a–c**) *XY* maximum intensity projections of the sample, with (**a**) showing the WF reconstruction from a single camera, (**b**) showing the MAPSIM reconstruction from one camera, and (**c**) showing the MAPSIM reconstruction from all four cameras. (**d–f**) *XZ* projections of the same data. (**g–i**) Zoomed-in view of the *XZ* projection from the region highlighted in (**d,j**), showing a 3D rendering of the four-camera MAPSIM reconstruction of the dataset, with the perspectives shown in (**c,f**) highlighted in green and magenta, respectively. Objective: UPLSAPO 60×/1.35 NA oil immersion.

**Figure 6.**
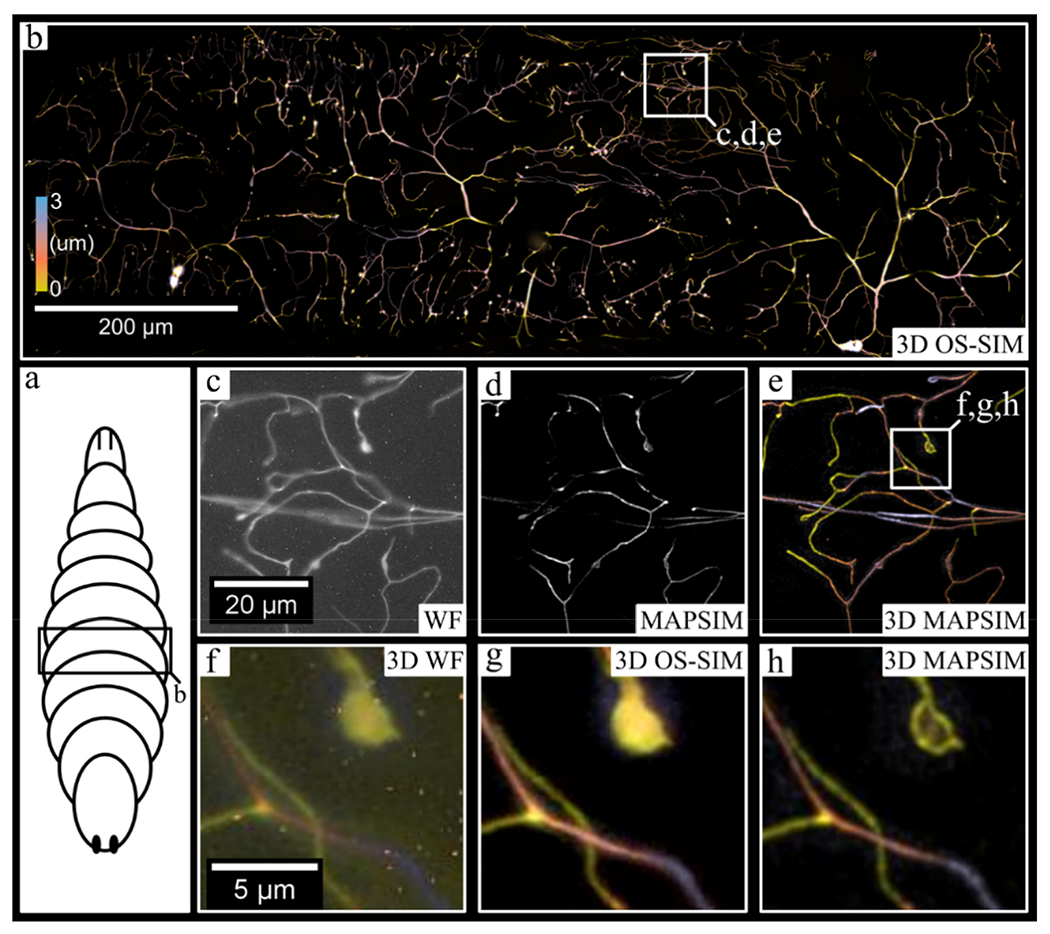
Large-FOV, 3D imaging of neurons in an abdominal segment of a *Drosophila melanogaster* larva. (**a**) (Generic illustration of a *Drosophila* larva, with the approximate imaging region labeled. (**b**) The entire 3D, stitched image produced from a mosaic of 3D images generated using the multicamera system. (**c–e**) The region indicated in (**b**), with (**c**) showing the WF reconstruction from a single camera, (**d**) showing the MAPSIM reconstruction from a single camera, and (**e**) showing the color-coded 3D image produced from MAPSIM reconstruction using all four cameras. (**f–h**) A comparison between SIM reconstruction methods on the region indicated in (**e**), using all four cameras: (**f**) WF; (**g**) OS-SIM; (**h**) MAPSIM. Note that hot-pixel removal was not performed on the images in (**c**,**f**) for demonstrative purposes. Objective: UPLSAPO 30×/1.05 NA silicone oil immersion.

To demonstrate the time-lapse imaging mode of our system, we imaged mitochondrial dynamics in HEP-G2 cells over 3 min. Hence, 3D images of a 88 × 88 × 1.6 μm region with an exposure time of 250 ms were acquired at 3 s intervals, resulting in 60 total frames, four of which are shown in Figure 4 (see Visualization 1 for a video containing all frames of the time-lapse). As evident in the figure, MAPSIM improves the resolution compared to the WF reconstruction and reduces out-of-focus light. In these images (Figure 4), the average SNR improved from 28.9 dB (WF) to 42.5 dB (MAPSIM), as measured by frequency-domain methods [44]. Due to the use of the four-camera detection system, all images were acquired with no physical *z*-movement of the sample and, with our SIM system, no moving parts during acquisition. To display the 3D information in Figures 4–6, the images were depth-coded using the isolum lookup table [45].

Next, we acquired a 130 μm thick 3D image of neurons in a GFP-labeled mouse brain sample at 60× magnification to demonstrate the 3D *Z*-stack imaging mode. This sample is much thicker than those typically imaged with SIM. Even though this 3D image has a size of 2048 × 1536 × 316 pixels, it was acquired in under 2 min using images with an exposure time of 100 ms per raw image. Due to the ability to capture four *z*-planes at once with the four-camera setup, the 316 *z*-planes in this image were acquired with just 79 movements of the *z*-stage. This image had 416 nm sampling in *Z* and 57.5 nm sampling in *XY*.

Next, we demonstrated the 3D mosaic imaging mode by imaging CD4-tdTomato labeled neurons in *Drosophila melanogaster* larvae (Figure 6). The 6957 × 10,151 pixel (~800 × 1150 × 3 μm) image was acquired in under 10 min, with each frame having an exposure time of 1500 ms. In these images (Figure 6), the average SNR improved from 18.3 dB (WF) to 45.1 dB (MAPSIM). For visualization purposes, this image was rotated, cropped, and color-coded to the region (~1000 × 300 × 3 μm) shown in Figure 6b. As shown in Figure 6d, the MAPSIM data from a single camera alone show improved resolution over the widefield reconstruction (Figure 6c), but MAPSIM’s optical sectioning prevents accurate visualization of neurons that deviate from the image’s narrow depth of field. When all four cameras are included (Figure 6e), the data not only provide useful depth information, but also have a larger effective depth of field than that of a single *z*-plane. This ability of the four-camera system to enhance the axial FOV of the montage substantially reduces complication during acquisition, as relatively flat samples can be entirely imaged without the need for autofocus systems or slide tilt/deflection correction.

**Figure 7.**
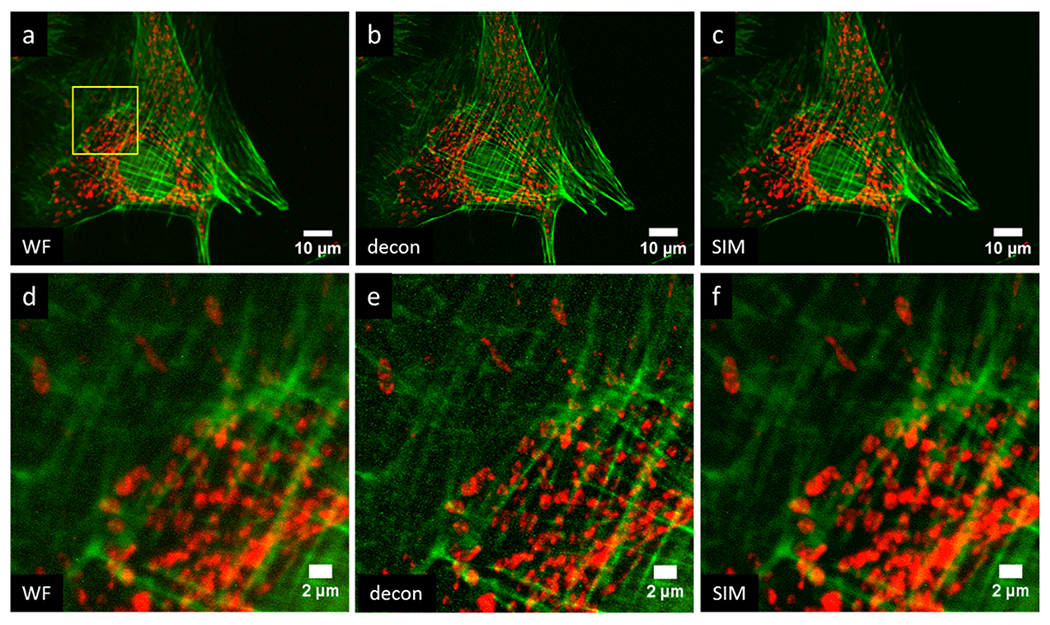
Four-camera, two-color imaging of actin (green) and mitochondria (red) in BPAE cells: (**a,d**) widefield; (**b,e**) Richardson–Lucy deconvolution; (**c,f**) MAPSIM. (**d–f**) Zoomed-in view of region indicated in (**a**). Objective: UPLSAPO 60×/1.35 NA oil immersion.

Lastly, we demonstrate multicolor imaging of a slide in which BPAE cells were labeled with MitoTracker red CMXROS and Alexa Fluor 488 phalloidin. These results are shown in Figure 7. Figure 7a,d show a widefield image, Figure 7b,e show the result of Richardson–Lucy deconvolution performed by DeconvolutionLab2 with 10 iterations and a theoretical PSF for each wavelength [46], and Figure 7c,e show the results of MAPSIM. In this particular case (Figure 7), MAPSIM did not improve the resolution beyond what was achieved with deconvolution. It can be seen that the deconvolution result, while slightly sharper, is also noisier than the SIM image.

**Figure 8.**
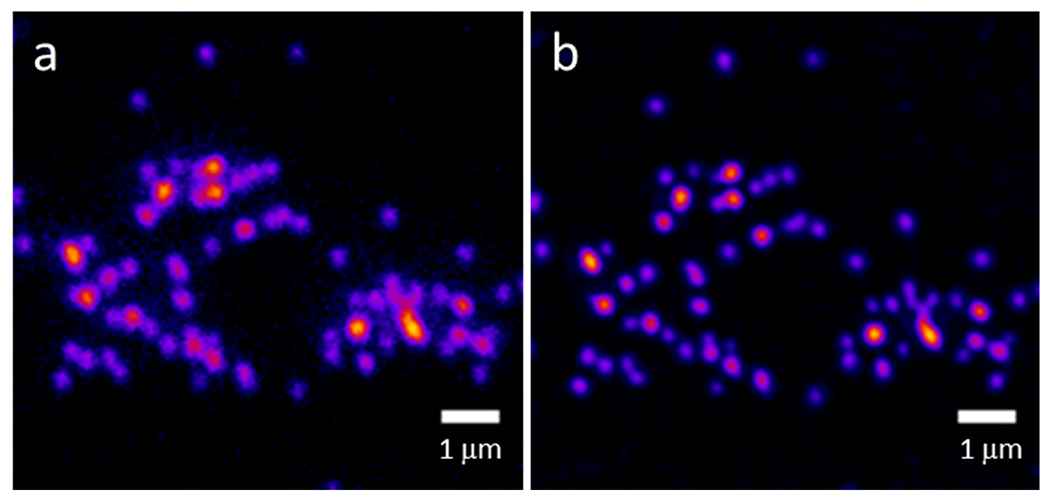
The 100 nm beads imaged with widefield (**a**) or MAPSIM (**b**) on one of the four cameras (camera 2). Objective: UPLSAPO 60×/1.35 NA oil immersion.

We measured the lateral resolution of the system using 100 nm fluorescent beads (F8800, ThermoFisher Scientific, Waltham, MA, USA) which were dried on a coverslip and overlaid with Prolong Glass (ThermoFisher Scientific, Waltham, MA, USA, η = 1.518). We imaged the beads using SIM illumination with all four cameras and the two objectives we used in this study (30× /1.05 NA silicone oil immersion and 60× /1.35 NA oil immersion). We used the same SIM parameters and camera positioning as used in Figure 4 (for 60×) and Figure 6 (for 30×). We used the Image J plugin ThunderSTORM to analyze the data [47], Using ThunderSTORM, we fit the bead images to an integrated Gaussian function using maximum-likelihood methods and calculated the full width at half maximum (FWHM) of each imaged bead. These results are shown in Table 2. Camera 4 is the most out of focus; therefore, on this 2D bead sample, the resolution measured with this camera is worse than the others. Figure 8 shows images of a field of 100 nm beads used in the resolution measurements for camera 2 using a 60× /1.35 NA oil immersion objective. Figure 8a shows the widefield image, while Figure 8b shows the MAPSIM result.

## 4. Discussion

While other implementations of multifocal plane microscopy have previously been demonstrated, our optical system is comparatively simple and versatile, and it requires no complex or custom optics. As a result, this system is substantially less expensive and easier to construct than alternative multifocal plane microscopy systems. The total cost of our four-camera detection system (including cameras, optics, and optomechanical components) was approximately one-quarter of the price of a typical sCMOS camera. Despite out) system’s simplicity and affordability, the image quality of our system is minimally compromised, as each camera’s image is relayed through a nearly 4*f* system (thus preserving telecentricity), and the beam-splitters in the optical system are in infinite space. Due to the recent improvements in CMOS image sensor technology, this simpler optical system was still able to obtain volumetric images of quality comparable to previous works, at four times the speed of a single-camera imaging system. We expanded upon previous multicamera systems by demonstrating a variety of the unique imaging modes possible with a multicamera system and super-resolution SIM reconstruction algorithms. Although some hot pixels were present in the raw image data, we were able to achieve satisfactory results by implementing a simple hot-pixel removal step in our image processing procedure.

Low excitation power is an important advantage of SIM compared to other super-resolution techniques, especially when imaging live cells where photobleaching and phototoxicity can be problematic. In all cases, the excitation power used here was less than 100 μW. Advantages to the use incoherent illumination in SIM include uniformity of the illumination across the field of view, removing the need for a mask to block unwanted diffraction orders. incoherent imaging of a microdisplay for pattern formation also means that the pattern spatial frequency in the sample plane does not depend on the wavelength of the light that is used.

However, this four-camera system does have drawbacks. Primarily, the intensity of light reaching each camera is one-quarter of that in a traditional single-camera system. This unavoidable reduction in light intensity on each detector necessitates longer exposure times (and thus slower imaging) on dim samples, such as the *Drosophila melanogaster* larvae and HEP-G2 cells imaged in this study. Although this downside would be substantial when using previous industrial CMOS technology, the high-sensitivity and low-noise image sensors used in this study were able to image such dim samples at reasonable exposure times. Additionally, this light intensity reduction is a downside common to all multifocal plane imaging techniques known to the authors with the exception of dual-objective microscopy in which two objectives image the sample from opposite sides onto two cameras [48]. The loss of light from performing multifocal plane imaging can compromise the system’s ability to acquire data at a rate faster than that of a single detection plane, but this does not impact the numerous other advantages of imaging multiple planes simultaneously.

Our method of defocusing the detectors away from the primary image plane means that the SIM pattern would be somewhat out of focus on three of the four cameras. This has the effect of demodulating the SIM pattern in the acquired images. The effect of this is shown in the Supplementary Materials (Figure S2).

Another issue present in our setup is the slight brightness variations between the images produced by the four cameras. Such brightness variations cause stripes in orthogonal projections of images acquired using the *Z*-stack mode, as visible in Figure 5i. These brightness variations are primarily due to tolerances in the transmission/reflection ratio of the beam-splitters used, which can result in intensity variations of ~5% between cameras. This issue could be resolved by using alternative beam-splitters with an improved transmission/reflection ratio tolerance. In this study, we were able to minimize these striping artefacts by normalizing the intensity of the image data between cameras after acquisition, although this method is imperfect (as seen in Figure 5i). The beam-splitters also introduce additional optical interfaces in the beam path, which is expected to degrade resolution because the surfaces are neither perfectly flat nor parallel. The resolution we achieved, shown in Table 2, is a bit worse than expected but similar to other multifocal plane imaging systems [29]. The optical aberrations introduced by the non-flat beam-splitter surfaces may also be responsible for the failure of MAPSIM to improve the resolution beyond that of deconvolution in the particular case of Figure 7.

Our four-camera system presents significant potential for future work. Firstly, the hot-pixel removal method used in this paper is functional, but primitive. Hot pixels that appear in the data which were not captured in the dark frame are not removed by this simplistic method. More sophisticated hot-pixel removal techniques, such as the one described in [49], could be used instead for higher-quality results. Current denoising algorithms could also be used to improve the results [50,51].

The four cameras in the system described here were used to image separate focal planes, but the detectors could also be used in slightly different configurations for an even wider variety of imaging types. By aligning the four cameras without defocusing the sensors, the cameras can quickly be triggered in succession to achieve combined frame rates four times higher than the cameras are individually capable of. This is particularly useful in applications where the frame rate is primarily limited by the readout time of the detector (such as brightfield imaging), rather than the exposure time (as is the case in SIM). Additionally, if the cameras are focused to the same plane but displaced laterally with the translation stages, the FOV of the detection system can be expanded by up to four times. This is particularly useful for cameras with small sensor sizes relative to the imaging circle of the microscope, such as the cameras in our setup. Lastly, if aligned laterally and kept focused, the four-camera setup could be used to generate high dynamic range (HDR) images by acquiring four images simultaneously with different exposure times, and then merging these images using HDR algorithms [52–54]. These potential uses of a multicamera system have previously been explored in the context of photography [55], but remain mostly novel in the field of microscopy.

Note that all of these additional imaging modes require no adjustment to the optics of our system; all that must be changed is the positioning and triggering synchronization of the cameras. As such, if the translation stages used to position the cameras were motorized, the four-camera detection system could be quickly and precisely reconfigured for a variety of uses. In the context of this work, such a motorized system could allow for quick adjustment of focal plane spacing, as well as automatically compensating when the microscope objective is changed. The system could also be easily reconfigured into the high-frame-rate or expanded lateral FOV modes previously discussed without tedious alignment. With sufficient image processing, a motorized system could even automatically align and focus the cameras.

To summarize, we presented in this study a highly flexible and practical multiplane imaging system using multiple cameras. This system is effective at quickly obtaining high-quality 3D fluorescence images using the three imaging modes explored in this paper, and we also proposed multiple additional imaging modes that could be explored with the same optical setup. The effectiveness, simplicity, and versatility of our optical system provides a promising approach for future implementations of multifocal plane imaging in fluorescence microscopy.

## Supplementary Material

supplementary material

video

## Figures and Tables

**Table 1.** Camera parameters.

Camera	FLIR Blackfly S	Andor Zyla 4.2+
**Part number**	BFS-U3-31S4M	ZYLA-4.2P-CL10
**Sensor type**	Sony IMX265	Andor Zyla
**Quantum efficiency**	62% at 525 nm	82% at 555 nm
**Read noise**	2.26 e^−^	0.9 e^−^
**Pixel size**	3.45 μm	6.5 μm
**Maximum frame rate**	55 FPS	100 FPS
**Readout method**	global shutter	rolling shutter
**Sensor size**	7.18 × 5.32 mm	13.3 × 13.3 mm
**Approximate price**	$530	$18,000

**Table 2.** Lateral FWHM measurements, nm.

	30×/1.05 NA		60×/1.35 NA	
	WF	MAPSIM	WF	MAPSIM
Cam 1	357.4	253.2	254.4	236.0
Cam 2	354.6	250.1	262.4	236.9
Cam 3	362.4	253.9	260.3	236.0
Cam 4	456.0	301.9	276.7	236.9

## Data Availability

Data are available by request from the authors.
