# Supplementary material for: Flexible Multiplane Structured Illumination Microscope with a Four-Camera Detector
Source: Photonics. Author manuscript; Available in PMC 2022 Aug 12. (PMC9373035; doi:10.3390/photonics9070501)
Supplement: supplementary material [file NIHMS1828296-supplement-supplementary_material.pdf]

photonics

Flexible Multiplane Structured Illumination Microscope with a Four-Camera Detector: Supplementary Information

KARL A. JOHNSON, DANIEL NOBLE, ROSA MACHADO, TRISTAN C. PAUL, AND GUY M. HAGEN*

UCCS BioFrontiers Center, University of Colorado Colorado Springs, 1420 Austin Bluffs Parkway, Colorado Springs, Colorado 80919, USA

**ghagen@uccs.edu*

1. Performance Comparison between Flir Backfly S 3154M and Andor Zyla 4.2+

To compare the noise performance of the FLIR industrial cameras used in the four-camera splitter configuration with the Andor Zyla 4.2+, images of an Argo-SIM calibration slide (Argolight, Pessac, France) were acquired with varying exposure times using each detector configuration. Additionally, to evaluate the impact of the four-way image splitter, the same experiment was performed with a single FLIR camera mounted directly on the microscope. To compare the signal-to-noise ratio (SNR) of a common feature across different cameras, the average intensity of the eighth-brightest line in the intensity gradient pattern was measured and normalized to the standard deviation of the noise level at that exposure time. Ten images were captured at each exposure time for each camera setup. The results are summarized in Figure S1.

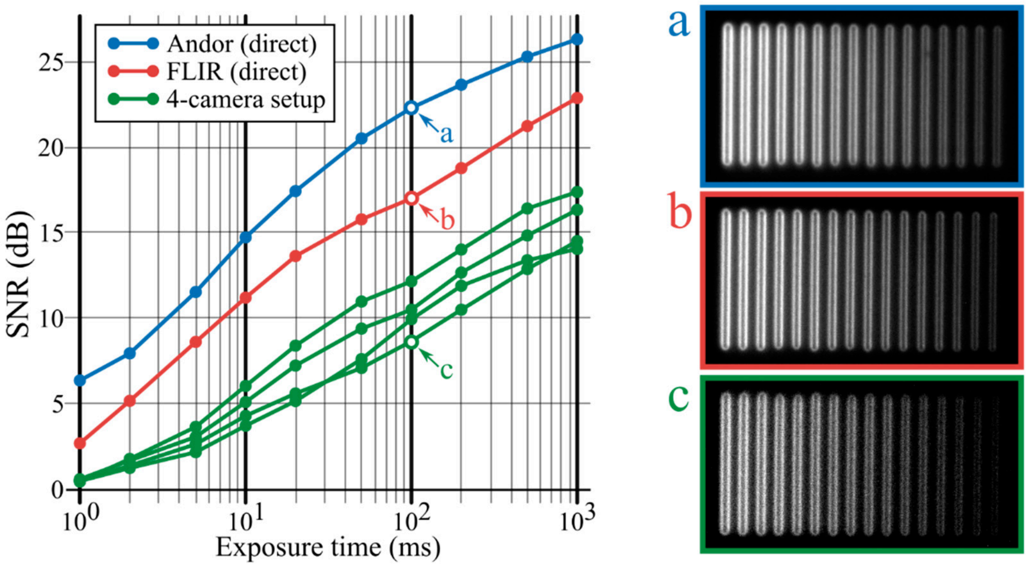

Figure S1. Log–log SNR measurements for the indicated camera configurations and qualitative image quality comparison. The plot to the left shows the SNR as the exposure time is varied for the Andor Zyla camera, FLIR camera directly mounted on the microscope, and FLIR camera as part of the four-camera detection setup. Images (a), (b), and (c) show an image of the Argolight intensity gradient structure captured with a 100 ms exposure time using the Andor Zyla, FLIR camera directly mounted on the microscope, and FLIR camera as part of the four-camera detection setup, respectively.

As is evident from the figure, the SNR of the FLIR cameras in the four-camera setup are on average 10 dB lower than that of the Andor Zyla on this particular image feature.

2. Modulation of the Illumination Pattern as a Function of Defocus Distance

To characterize the effects of sensor defocus on the contrast of the SIM illumination pattern, we imaged a uniform thin fluorescent layer with SIM patterns of varying spatial frequencies and recorded the modulation as a function of sensor defocus. The modulation was calculated from the intensity data as (max − min)/(max + min). A summary of the results is shown in Figure S2.

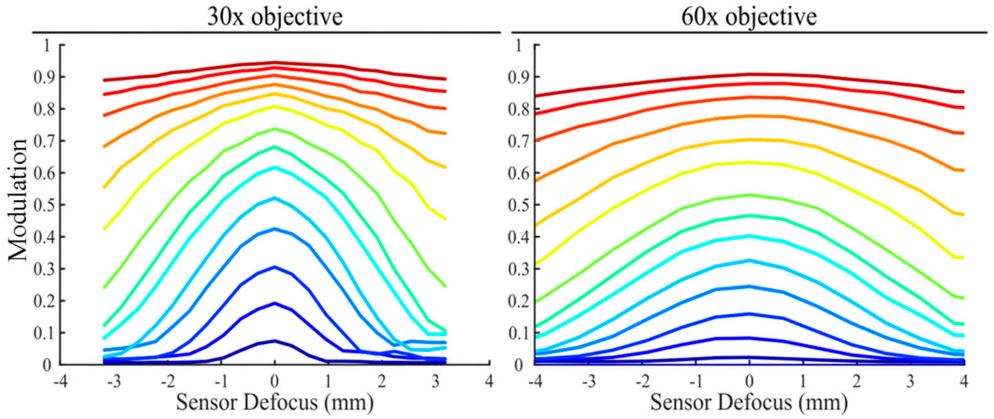

Line color														
SLM pattern per. (px)	36	26	20	16	13	11	9	8	7	6	5	4	3	2
30x/1.05 norm. freq.	.032	.044	.055	.072	.089	.105	.128	.144	.165	.192	.231	.288	.385	.577
60x/1.35 norm. freq.	.050	.069	.090	.138	.128	.163	.199	.224	.256	.299	.359	.449	.598	.897

Figure S2. Measured SIM pattern modulation as a function of sensor defocus for 30×/1.05 NA and 60×/1.35 NA objectives. Individual colored lines on each plot represent the modulation versus sensor defocus for each illumination pattern. The legend at the bottom of the figure indicates the spatial period of each pattern on the SLM, as well as the corresponding normalized spatial frequency of the patterns for each objective.

Although sensor defocus has the anticipated impact of reducing the modulation of higher-spatial-frequency SIM patterns, Figure S2 demonstrates that the adverse effects of defocus on the modulation of the illumination can be combated simply by selecting a lower-spatial-frequency SIM pattern.

3. Acquisition Parameter Summary

Table S1. Summary of acquisition parameters.

Parameter	Mitochondria (live) (Figure 4)	Mouse brain (Figure 5)	*Drosophila* larva (Figure 6)
**SIM pattern period (pixels)**	11	12	12
**SIM pattern normalized spatial frequency**	0.163	0.150	0.096
**Fluorophore**	Mitotracker	eGFP	tdTomato
**Exposure time**	250 ms	100 ms	1500 ms
**Ex/Em wavelengths (nm)**	578/599	490/509	555/581
**Excitation power**	92 μW	36 μW	88 μW
**Objective**	60×/1.35 oil	60×/1.35 oil	30×/1.05 silicone oil
**Backprojected**	57.5 nm	57.5 nm	115 nm

**Pixel size**	

**Parameter**	**Actin and mitochondria (fixed) (Figure 7)**
**SIM pattern period (pixels)**	12
**SIM pattern normalized spatial frequency**	0.150
**Fluorophore**	Alexa Fluor 488 and MitoTracker Red
**Exposure time**	100 ms for both
**Ex/Em wavelengths (nm)**	498/520 and 578/599
**Excitation power**	36 μW (blue ex) / 88 μW (green ex)
**Objective**	60×/1.35 oil
**Backprojected Pixel size**	57.5 nm
